# Report of a Case of Tubal Pregnancy within the Wall of the Uterus

**Published:** 1912-10

**Authors:** C. W. Hennington

**Affiliations:** Surgeon to Rochester State Hospital. Surgeon to Out Patients; Rochester General Hospital


### Report of a Case of Tubal Pregnancy Within the Wall of the Uterus.

By C. W. HENNINGTON, B.S., M.D.
Surgeon to Rochester State Hospital. Surgeon to Out Patients
Rochester General Hospital.

THE patient is a young woman of 29 who has been married about five years and has one child. The labor and puerperium seems to have been normal in every respect and there is nothing in the general history or pelvic history that is worthy of note. Although three years have elapsed there had been no subsequent pregnancy. Her menstrual history shows a regular twenty-eight or twenty-nine day interval. The periods lasted five to seven days. The flow was normal in amount and not associated with any unusual pain. She never took to her bed.

The present illness may probably be dated from March 7, 1912, when she failed to menstruate. She recalls no symptoms at this time and supposed that she perhaps was pregnant. Two weeks later on March 21, she began with a profuse flow which lasted for at least nine or ten days. She was not ill and continued actively doing her house work. For the next months she menstruated regularly about April 22, May 22 and June 22. Each time there was the usual flow. However during this interval there were occasional days when a little bleeding occurred sufficient to slightly soil the clothing. During the last month she felt slight pain in the lower abdomen.

The actual illness commenced in the morning of June 28th with intense abdominal pain, general at first but soon located in the pelvis and the right iliac fossa. Morphia was required repeatedly. There was no vomiting at this time. The bowels moved frequently. Temperature and pulse were said to be only slightly, if at all, above normal. On the following day patient had vomited once, temperature showed no change, but the pulse rose to 120. She was brought to Rochester for operation that afternoon. On admission temperature was 100, pulse 120 and respiration 24.

Operation. June 29, 1912. 6 P. M. Low mid-line incision. Considerable free fluid, cloudy and with flakes of fibrin. Peritoneal surfaces red, dull and agglutinating. Appendix persented free in the wound and was removed. The uterus was soft and had a nodule in the region of the right horn which had perforated and was discharging pus into the peritoneal cavity. A hurried supra-vaginal hysterectomy with both tubes but only the right ovary was quickly done. The pelvis was free of any blood or inflammatory masses or adhesions. No cultures were taken. A large rubber drainage tube was placed through the lower end of the incision to the bottom of the cul-de-sac. Wound closed in usual manner.

Post-Operative History. Extreme Fowler's position and continuous saline per rectum. Uneventful and rapid recovery. Left Rochester for her home in three weeks and is now in perfect health.

Pathological Description of the Specimen. The uterus itself is of about normal size. On the right side and right fundus its shape is distorted by the presence of a nodule situated within the uterine wall in the region of the cornu. On the posterior and upper surface of this right end of the fundus is a perforation of the nodule from which pus is escaping. The tube is normal in appearance and joins the uterus at the lower outer surface of the nodule. The right round ligament is seen near the tube also on the lower outer surface of the nodule. The perforation just referred to is some 2cm. away and on the upper surface of the nodule.

On cutting the uterus open in the usual manner, the walls are found to be of normal thickness, that is nearly 2cm. The cavity is lined with bloody thickened endometrium. The triangular appearance of the cavity is not disturbed but just beyond the right cornual extension of that cavity and between it and the right tube is a spherical mass 2½cm. in diameter. This mass is surrounded on the upper and outer sides by uterine wall slightly less than ½cm. in thickness, but toward the cavity of the uterus it is separated from that cavity by only the thinnest layer of uterine wall, in fact no thicker than the endometrium itself. A probe can be passed down the tube into the interstitial portion within the wall of the uterus and approaches the mass directly, though it does not freely pass into it. From the aspect of the uterine cavity it is noted that the right cornual extension of that cavity points directly toward the mass and is separated from it only by the thin tissue referred to above. Thus the anatomical origin of the mass is shown to have been in the interstitial portion of the tube.

The mass itself consists of a thickened organized lining, now of the dark greenish to whitish hue, which can be separated easily from the cavity which it had produced in the uterine wall. The center of the mass is of a grumous friable content of irregular consistency and of dark color mixed with thick pus. Microscopic examination revealed the presence of placental tissue and confirmed the diagnosis of pregnancy.

Conclusion. Thus we have a case of tubal pregnancy in the interstitial portion of the tube, that is, in that portion of the tube which is burrowing its way through the wall of the uterus. Stated in a paradoxical way, we have an extra-uterine pregnancy within the uterus. This is the rarest variety of tubal pregnancy. It constituted only 3% of the 1324 cases collected by Rosenthal and only 1 of the 77 cases carefully described by Martin. From a consideration of the history and findings of this case it is evident that the course of this particular instance is of especial interest and constitutes the sole reason for publication.

